# A prospective study of shared decision-making in brain tumor surgery

**DOI:** 10.1007/s00701-022-05451-z

**Published:** 2022-12-28

**Authors:** Severina Leu, Julian Cahill, Paul L. Grundy

**Affiliations:** 1 Department of Neurosurgery, Wessex Neurological Centre, University Hospital Southampton, Southampton, Hampshire UK; 2 Department of Neurosurgery, University Hospital Basel, Spitalstrasse 21, 4031 Basel, Switzerland; 3 Department of Neurosurgery, Royal Hallamshire Hospital, Sheffield, South Yorkshire UK; 4 The National Centre for Stereotactic Radiosurgery, Sheffield, South Yorkshire UK

**Keywords:** Decision aid, Glioma, Metastases, Shared decision-making, Treatment options

## Abstract

**Purpose:**

Shared decision-making (SDM) is a key tenet of personalized care and is becoming an essential component of informed consent in an increasing number of countries. The aim of this study is to analyze patient and healthcare staff satisfaction with the SDM process before and after SDM was officially introduced as the standard of care. Decision grids are important tools in the SDM process, and we developed them for three different types of intracranial tumors.

**Methods:**

This prospective study was conducted in a high-volume neuro-oncological center on all consecutive eligible patients undergoing consideration of treatment for intracranial glioma and metastases. Twenty-two patients participated before and 74 after the introduction of SDM. Six and 5 staff members respectively participated in the analysis before and after team training and the introduction of SDM. The main outcome was patient and healthcare staff satisfaction with the SDM process.

**Results:**

Patients reported high satisfaction with the SDM process before (mean CollaboRATE score 26 of 27 points) and after (mean CollaboRATE score 26.3 of 27 points, *p* = 0.23) the introduction of SDM. Interestingly, staff attitude toward SDM improved significantly from 61.68 before to 90.95% after the introduction of SDM (*p*-value < 0.001). Decision grids that were developed for three different types of intracranial tumors are presented.

**Conclusions:**

Team training in SDM and the introduction of techniques into daily practice can increase staff satisfaction with the SDM process. High levels of patient satisfaction were observed before, with a non-significant increase after the introduction of SDM. Decision grids are an important tool to facilitate the conveyance and understanding of complex information and to achieve SDM in daily clinical practice.

**Supplementary information:**

The online version contains supplementary material available at 10.1007/s00701-022-05451-z.

## Introduction

Shared decision-making (SDM) is one of the key components of personalized care, recognizing that patients and families increasingly want, and expect, to be involved in healthcare decisions. In delivering SDM, the patients’ views, values, preferences, and beliefs are considered when making a decision together with their clinical team based on what matters most to them. It is an honest conversation about reasonable competing options, ensuring the patient understands all the risks and benefits of individual treatment options.

The increasing importance of SDM for the United Kingdom (UK) healthcare practice and especially cancer care is shown by the rise in the official guidance and guidelines. In 2015, a Supreme Court Judgment in the case of Montgomery v Lanarkshire Health (2015) UKSC 11 changed the law on medical consent in the UK healthcare system. This decision established that consent to medical treatment requires clinicians to determine and explain what might be materially important to a patient, shifting to a patient-centered model and away from medical paternalism. SDM has, thus, become a legal requirement in the UK and clinicians now must take “reasonable care to ensure that the patient is aware of any material risks involved in any recommended treatment and of any reasonable alternative or variant treatments” [22, 24]. SDM is also an important topic for the National Institute for Health and Care Excellence (NICE) and an official NICE guideline about shared decision-making was published on 17th June 2021 [26].

SDM was first mentioned in 1982 in Washington in a president’s commission [41]. Despite a huge amount of implementation effort and ethical and policy support, SDM has not yet become the standard of care in cancer care in the United States (US) [16, 33, 36].

SDM can be achieved and improved using certain conversation techniques and schemes. These techniques need to be learned, and healthcare staff need to be trained to be adequately integrated into daily clinic routine and applied as the standard of care. Theoretical followed by practical team training seems to be a helpful tool to reach these goals. Data about the impact of SDM on healthcare professionals in the literature is sparse, as most of the outcome research about SDM focuses on the well-being of patients. To our knowledge, this is the first study examining the impact of SDM on staff in neuro-oncology.

Joseph-Williams et al. looked at the implementation of SDM into the National Health Service (NHS) in 2017 [21] while analyzing the lessons learned from the MAGIC program (Making Good decisions In Collaboration) in 2010 [17, 18]. They felt that role play-based training worked better than theory-heavy presentations for awareness and training of staff. Patient activation campaigns such as “ask 3 questions” can help patients to find out what matters to them and encourage more involvement in the decision-making process. They found it difficult to measure the success of SDM implementation; however, they felt that the three-item CollaboRATE measure that was used in over 40 studies worldwide shows promise in overcoming these problems [2, 3, 12]. It seems to be important that SDM is used and supported by all members of the clinical team and not only by doctors. Their conclusion was that successful implementation of SDM relies on a combination of interventions supporting the organization, clinicians, and patients and that organizational support and local ownership are vital for engagement [21]. Surveys have shown that patients want to be more involved than they currently are in decisions concerning their own health and decisions about treatment [8, 28]. Mulley et al. found that SDM can create a new relationship between patients and clinicians that is based on partnership. Moreover, SDM has the potential to enhance allocative efficiency and reduce unwarranted clinical variation [25].

The aim of this study was to analyze patient and healthcare staff satisfaction with the SDM process in brain tumor surgery. This was done before and after team training in SDM was conducted and SDM was officially introduced as the standard of care. The CollaboRATE score was used to analyze patient satisfaction, and the attitude of healthcare staff members was measured using the AquA questionnaire [2, 3, 12, 27]. During this study, three decision aids for three different types of brain tumors were developed that are used in daily clinical practice as an important tool to achieve SDM.

## Methods

### Participating patients

The study was conducted in a high-volume neuro-oncological center on all consecutive eligible patients coming to their first clinic visit and undergoing consideration of treatment for high-grade glioma (HGG), low-grade glioma (LGG), and metastases.

Twenty-two patients participated in the study period before the team was trained in SDM (further referred to as group 1). This study period lasted from November 2017 to January 2018. Seventy-four patients participated in the study period after team training and the introduction of SDM (further referred to as group 2), and this period lasted from January 2018 until February 2019.

Informed consent from all study participants was obtained before inclusion in the study. This study was conducted as part of a program from NHS England called Commissioning for Quality and Innovation (CQUIN) in the first cycle of a Plan-Do-Study-Act (PDSA) process [7].

### Participating carer team

The neurosurgical neuro-oncological team at the University Hospital Southampton during the study consisted of 3 neurosurgeons and 3 clinical nurse specialists (CNS). One of the neurosurgeons was a consultant, and 2 were consecutive senior neurosurgical post certificate of Completion of Training (CCT) fellows for the years 2017 and 2018. The CNS service was exclusively focusing on brain tumor patients, and each of the three CNS had at least 10 years of experience in working with brain tumor patients.

### Team training in SDM

The lead neurosurgeon of the team went to 2 training days with NHS England, a combination of classroom teaching, role play, discussion, and presentations by patients. He then cascade-trained the others using front-of-class teaching to convey the basics of SDM, followed by role plays where routine clinic situations were simulated. Further local on-site training was carried out by the lead neurosurgeon with the team. Thereby, techniques and illustrations were integrated into daily clinic use and maintained as the standard of care afterwards.

### Development of decision grids

The grids were developed based on the up-to-date National Institute for Health and Care Excellence (NICE) guidelines [29] and the treatment options available at our unit in line with the guidance for Grid creation from Option Grid™ [37]. Option Grid™ [30] provides a template for a summary table, using one side of an A4 sheet to enable a rapid comparison of options, using questions that patients frequently ask (FAQs) and are designed for a face-to-face clinical encounter [14]. The grids were customized and written to a reading age of 12 years.

We created decision grids for three types of intracranial tumors (HGG, LGG, and metastases), and for each tumor, three different treatment options were given (HGG: resection, biopsy, or Best Medical Care (BMC), LGG: resection, biopsy, or active surveillance, Metastases: resection, stereotactic radiosurgery/radiotherapy, or BMC). The choices and content were taken from our routine conversations with risks and benefits derived from our own results and those published in the literature. The level of risk can be adjusted depending on an individual case basis. We used NHS England quality improvement methodology such as PDSA (Plan-Do-Study-Act) cycles [32], and after grid creation, we searched input from a patient charity representative from Braintrust [5].

Once created, the decision grids were further refined over time and updated at regular intervals.

Decision grids facilitate the SDM process [14, 15, 23, 31, 38, 42–44]. They can help in simplification and stratification of the amount of information given to patients in difficult situations. The grids can be used as a help to structure the conversation about these options with patients in clinic.

## Analysis

### Patients attitude to SDM before and after training and the introduction of SDM was assessed with the CollaboRATE questionnaire [1–3, 12]

**Fig. 1:**
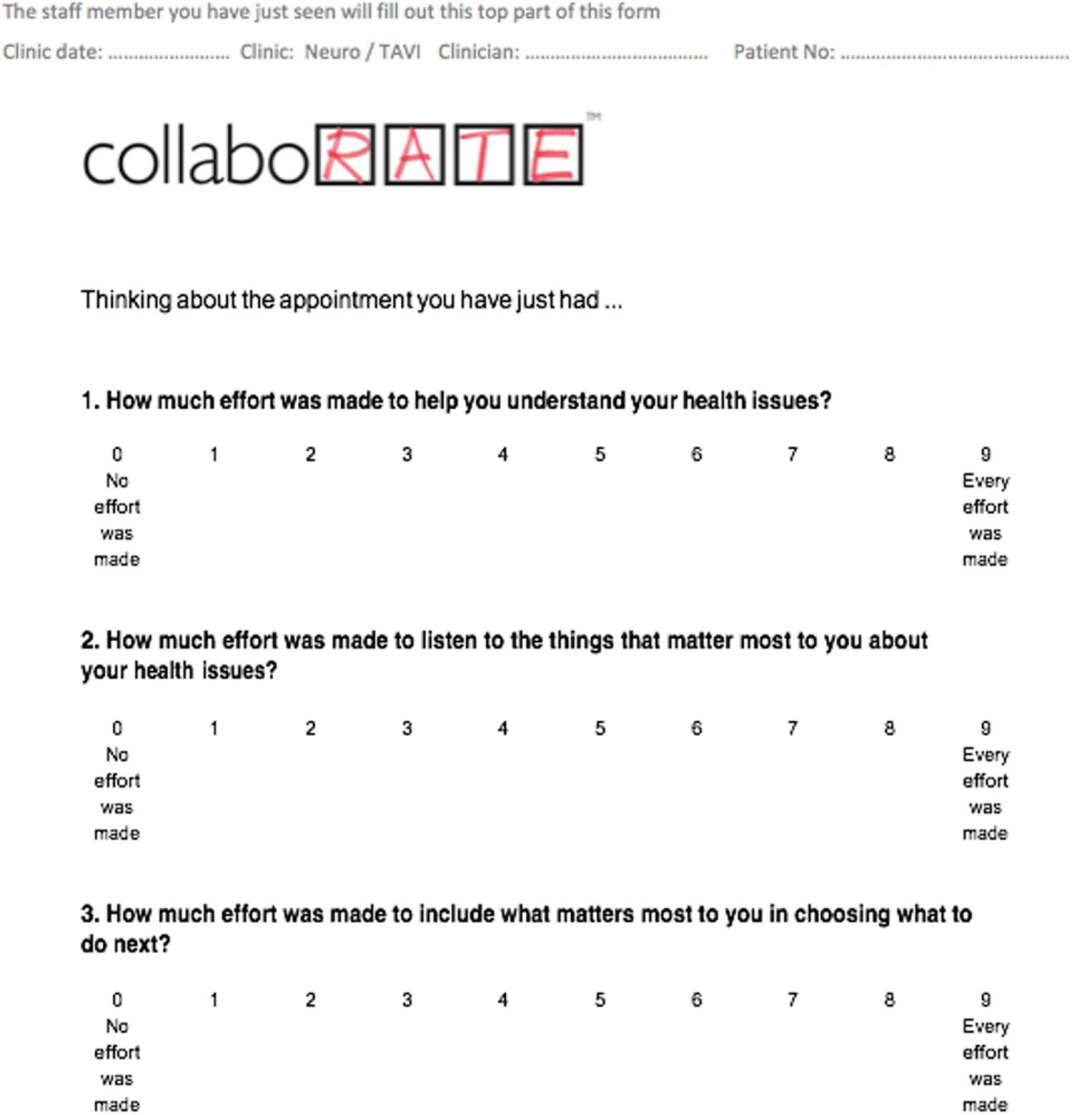
Questions contained in CollaboRATE questionnaire and scoring options for individual questions between 9 (every effort was made) and 0 (no effort was made). The total score can range from 27 to 0 points [2, 3, 12]

The collaboRATE questionnaire (Fig. 1) is a patient-reported measure containing 3 items:

- How much effort was made to help them understand their health issue
- How much effort was made to listen to the things that matter most to them about their health issue
- How much effort was made to include what matters most to them in choosing what to do next

The patient can give scores between 0 (no effort was made) and 9 (every effort was made) for each of these points, and a total score can then range between 0 and 27 points. A higher score means a better experience [1–3, 12].

### Clinicians attitude to SDM before and after training and the introduction of SDM assessed with the AQuA questionnaire [27]

**Fig. 2:**
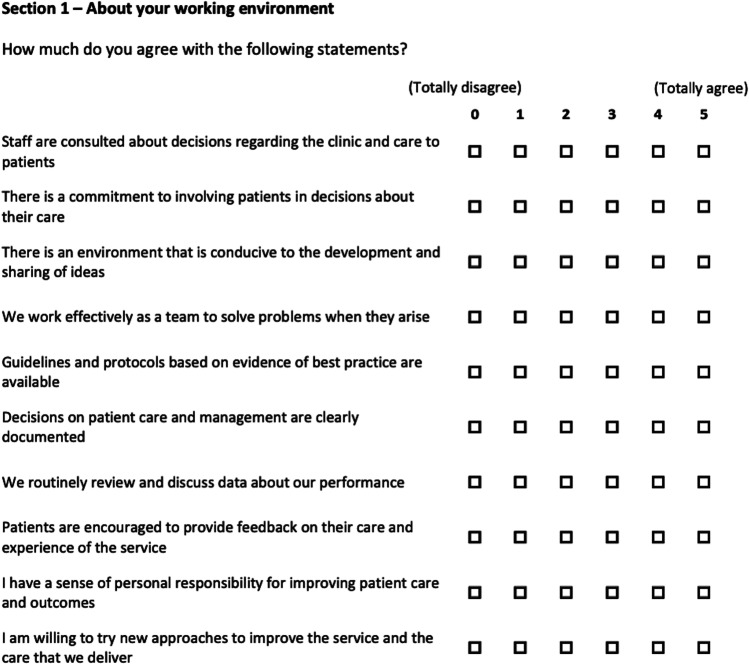
Sect. 1 with questions 1 to 10 of the AQuA questionnaire. For every question, a score between 5 (totally agree) and 0 (totally disagree) can be given, leading to a total score between 50 and 0 [27]

**Fig. 3:**
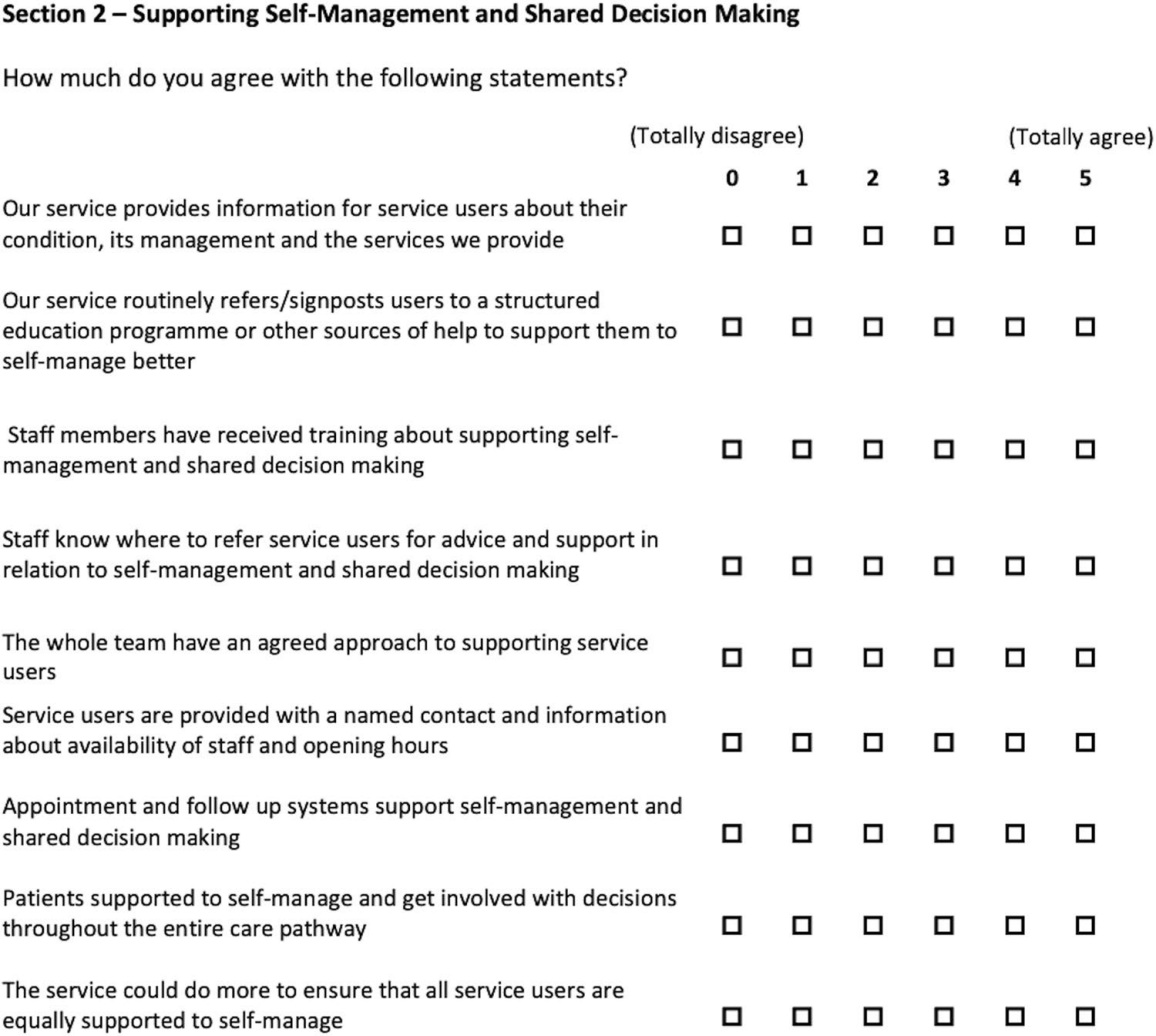
Sect. 2 with questions 11 to 19 of the AQuA questionnaire. For every question, a score between 5 (totally agree) and 0 (totally disagree) can be given, leading to a total score between 45 and 0 [27]

The AQuA questionnaire consists of two sections containing 10 and 9 questions, respectively. The first section focuses on the work environment and teamwork (Fig. 2). The second section elucidates how much self-management of patients and SDM is supported (Fig. 3). For every question, a score between 0 (totally disagree) and 5 (totally agree) can be given, leading to possible total scores between 0 and 95 points [27].

### Data collection and statistical analysis

Data from patients was collected after their first clinic appointment where options were presented and discussed. Data from staff members was collected at two different time points. The first time point was before team training and the introduction of SDM, after the first 22 patients have been included in the study, and the second time point was after team training and the introduction of SDM and after the 74 patients had been included in the study. Both data collections from patients and staff members were anonymized. Summary statistics were used to outline baseline characteristics (Table 1). Scores of the CollaboRATE questionnaire and the AquA questionnaire were analyzed using bivariable analysis, and a *p* value of < 0.05 was considered significant. Multivariable analysis was done for the CollaborRATE questionnaire scores using multiple analysis of variance (ANOVA), including the variables gender and tumor types (Table 2). Post hoc analyses were done for the variable tumor type using the Bonferroni correction. All statistical analyses were performed using SPSS [20].

**Table 1:** Baseline characteristics of patients in group 1 and group 2

	Before SDM introduction Group 1	After SDM introduction Group 2	*p* value
Total	22	74	
Mean age in *y*	58.0 (19.4–80.5)	63.7 (30.4–79.2)	0.10
Male (in %)	68.2	58.1	0.40
HGG	12	37	0.71
LGG	4	6	0.17
Metastasis	4	20	0.40
Missing histology	2	11	0.49
Death rate in %	59.1	66.2	0.71

**Table 2:** Multivariable analysis of CollaboRATE score results including the factors intervention (team training and introduction of SDM), tumor type (HGG, LGG, metastasis), and gender. None of the variables did show a significant impact on CollaboRATE score values

Source	Type III square sum	*df* value	Mean of squares	*F* value	*p* value	Partial eta^2^
Corrected model	42.034^a^	11	3.821	0.504	0.894	0.072
Constant term	25,056.673	1	25,056.673	3306.799	< 0.001	0.979
Intervention	3.844	1	3.844	0.507	0.479	0.007
Tumor type	6.399	2	3.200	0.422	0.657	0.012
Gender	0.953	1	0.953	0.126	0.724	0.002
Intervention * tumor type	0.610	2	0.305	0.040	0.961	0.001
Intervention * gender	2.041	1	2.041	0.269	0.605	0.004
Tumortype * gender	15.046	2	7.523	0.993	0.376	0.027
Intervention * tumor type * gender	3.005	2	1.502	0.198	0.821	0.006
Fault	537.990	71	7.577			
Total	57,157.000	83				
Corrected overall variation	580.024	82				

^a^*R* square = 0.072 (corrected *R* square =  − 0.071)

## Results

### Patients baseline characteristics

Among the 22 patients in group 1, 12 were diagnosed with HGG, 4 with LGG, and 4 with metastases after final histology (2 without histology). The 2 patients without histology chose BMC, 7 patients chose biopsy, and 13 patients chose tumor resection from the available treatment options. Age ranged from 19.4 to 80.5 years with a mean age of 58.0 years. 68.2% of these patients were male (15/22). Thirteen of these 22 patients have died in the meantime (59.1%).

The 74 patients in group 2 consisted of 37 patients with HGG, 6 patients with LGG, and 20 patients with metastases (11 with other/no histology). From the 11 patients with other/no histology, 9 patients chose BMC as a treatment option, and 2 underwent biopsy showing a different histology (both primary CNS lymphoma). From the remaining patients, 16 opted for biopsy, and 47 patients opted for tumor resection from the available treatment options. Age ranged from 30.4 to 79.2 years with a mean age of 63.7 years. 58.1% of these patients were male (43/74). Forty-nine of these 74 patients have died in the meantime (66.2%).

None of the baseline characteristics significantly differed between the two groups (Table 1).

### Patients attitude to SDM before and after training and the introduction of SDM assessed with the CollaboRATE questionnaire [2, 3, 12]

**Fig. 4:**
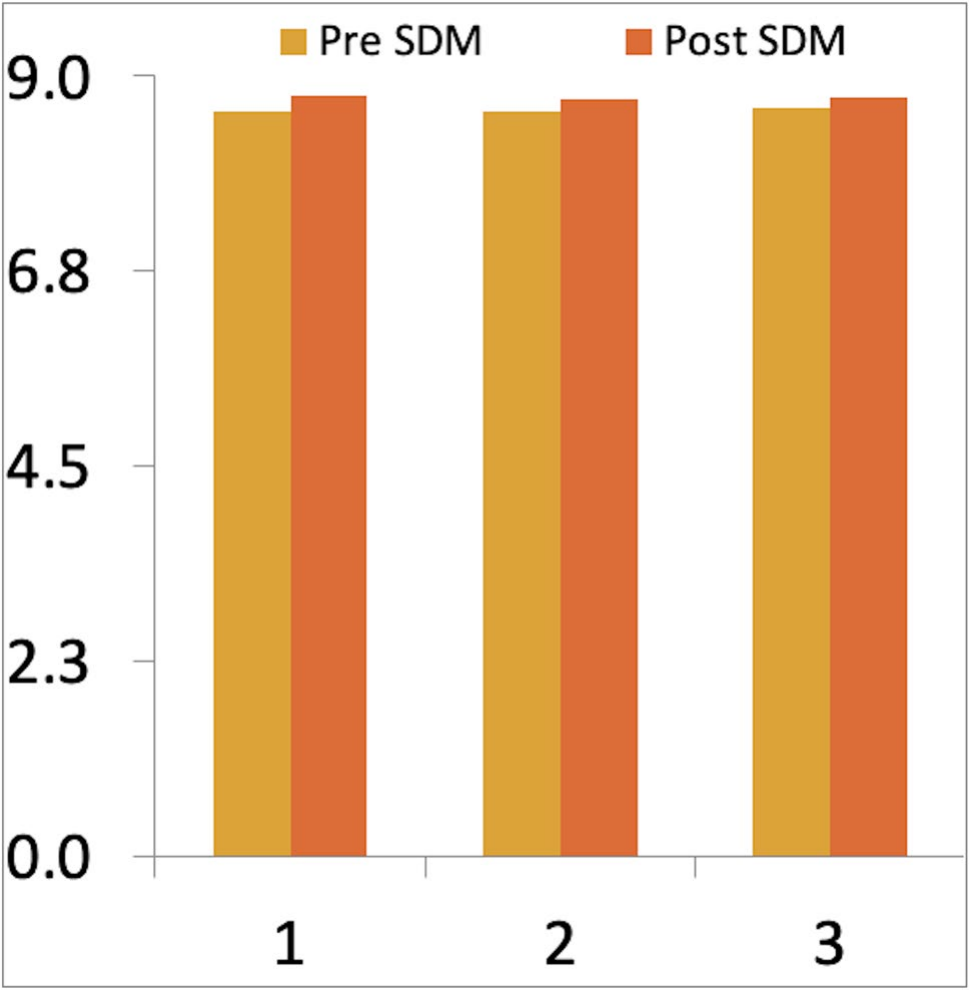
Average patient scores to questions 1 to 3 of CollaboRATE questionnaire before and after SDM introduction

Twenty-two patients completed CollaboRATE before and 74 after team training and the introduction of SDM. The CollaboRATE score was already high before the introduction of SDM with a mean score of 25.82/27 (range 15–27) and increased non-significantly to a mean score of 26.27/27 (range 11–27) after SDM was introduced (*p*-value = 0.23) (Fig. 4).

A multivariate analysis including factors such as tumor type (HGG, LGG, metastasis) and gender did not show a statistically significant difference (Table 2).

### Clinicians attitude to SDM before and after training and the introduction of SDM assessed with the AQuA questionnaire[27]

**Fig. 5:**
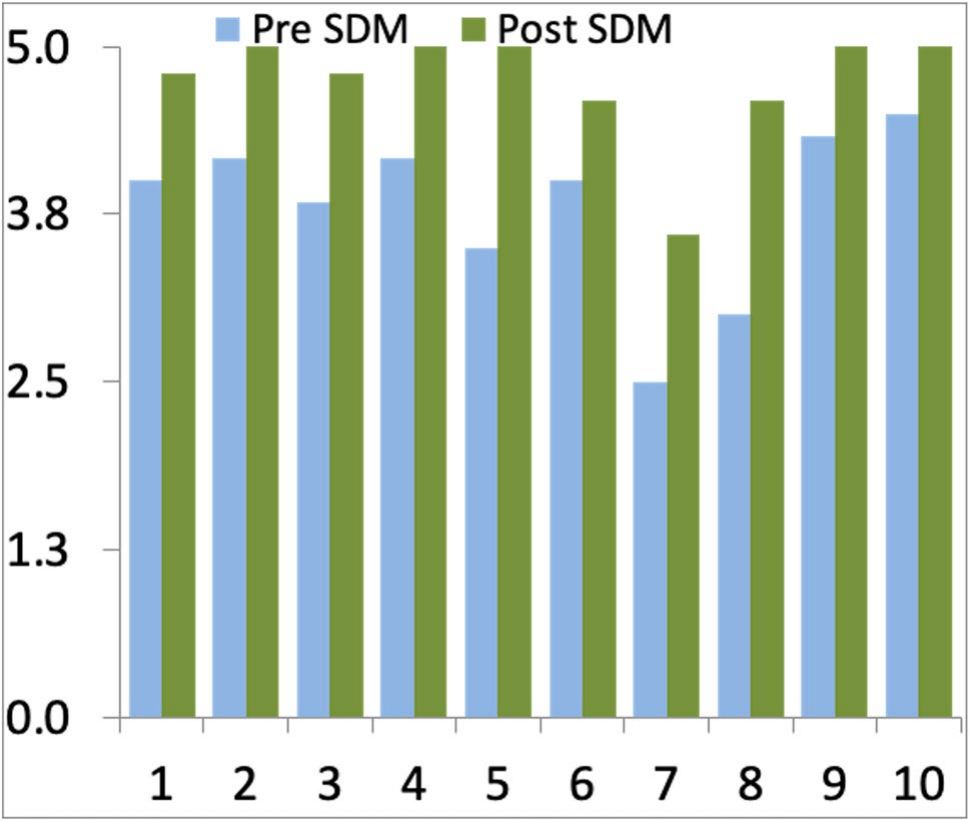
Total/average staff scores in questions 1 to 10 of the AQuA questionnaire before and after SDM introduction

**Fig. 6:**
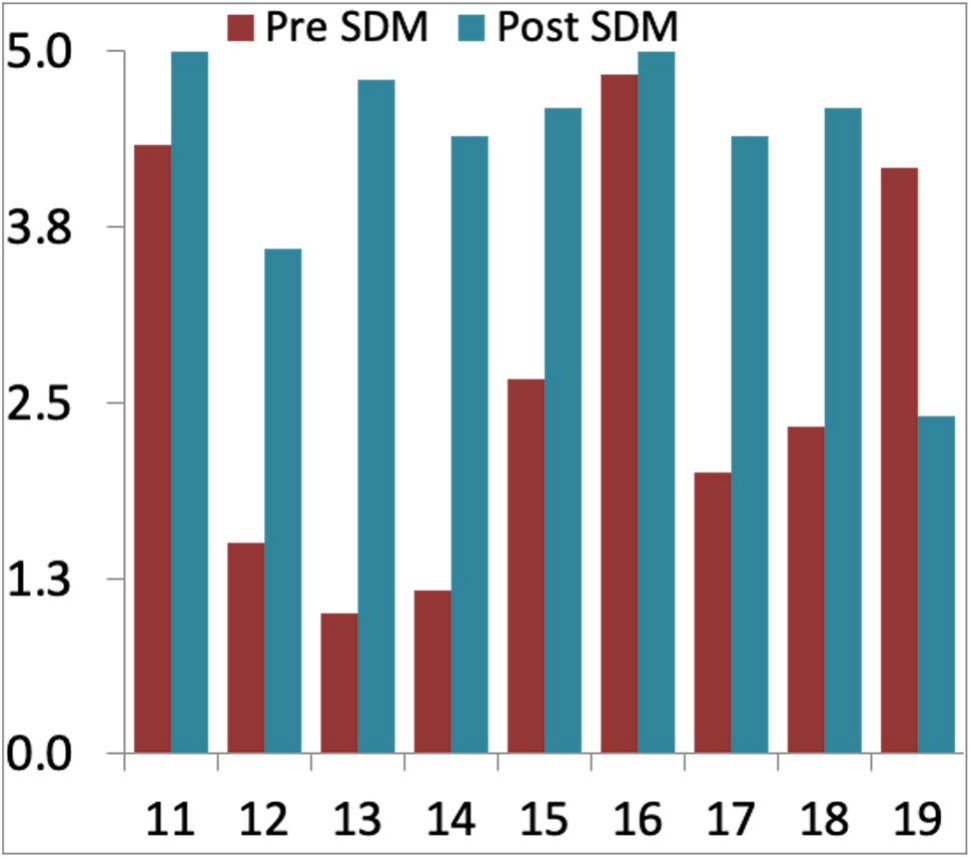
Total/average staff scores in questions 11 to 19 of the AQuA questionnaire before and after SDM introduction

Six out of 6 staff members completed the pre-introduction survey, and 5 out of 6 staff members completed the post-introduction survey. Reached total scores changed from 61.68 before to 90.95% after the introduction of SDM (*p*-value < 0.001). In particular, scores of Sect. 1 changed from 76.00 before to 94.80% after SDM was introduced (*p*-value < 0.001) (Fig. 5), and Sect. 2 scores changed from 45.78 before to 86.67% post-introduction (*p*-value < 0.001) (Fig. 6). Every question can be scored by a maximum of 5 points. Mean score values given to each question did significantly change from 3.08 before to 4.55 post-introduction (*p* < 0.001).

### Decision grids

Decision grids for three different types of intracranial tumors were developed showing the three most appropriate treatment options. For surgical options, the most common risks are given in % values, and these values can be adjusted depending on the individual case. If the multidisciplinary team (MDT) meeting determines that a tumor is not amenable to resection or is unsuitable for stereotactic radiosurgery, these options were not presented to the patient.

### Decision grid HGG

Detailed information about treatment options for craniotomy and tumor removal (+ / − chemo/radiotherapy), biopsy (+ / − chemo/radiotherapy), and BMC is listed in parallel (Supplementary Table 1).

### Decision grid LGG

Detailed information about treatment options for craniotomy and tumor removal, biopsy, and active surveillance is listed in parallel (Supplementary Table 2).

### Decision grid metastases

Detailed information about treatment options for craniotomy and tumor removal, stereotactic radiosurgery/-therapy, and BMC is listed in parallel (Supplementary Table 3).

### Current SDM process at our institution resulting from the findings of this study

Concordance with the NICE guidelines [29] after radiological diagnosis of glioma cases are discussed in the weekly MDT meeting. The MDT consists of neurosurgeons, neuroradiologists, neuropathologists, neuro-oncologists, neuropsychologists, and neuro-oncology specialist nurses. The surgical expertise in the MDT has access to awake craniotomy with language and other appropriate functional monitoring, expertise in intraoperative neurophysiological monitoring; access to neuroradiological support; and access to intraoperative image guidance.

Patients will then come to the outpatient clinic, usually accompanied by their next of kin. Before the clinic appointment, they are often poorly or not informed about their diagnosis, treatment options, and prognosis. However, there are some patients that have been well informed before by other doctors or by self-research, e.g., on the Internet.

At the beginning of the clinic visit, we determine what matters most to the patient and explore their interests, beliefs, experiences, and approach to risk. In the future, this will be done prior to the first clinic visit over the online tool “MyMedicalRecord,” where the patients will be asked to fill in specific questionnaires concerning these questions.

During the appointment, patients are informed in a compassionate manner about the nature of their tumor, the treatment options, and the expected prognosis with the different treatment options. If they have a malignant tumor, they are told that the tumor is not curable and limits their life expectancy. Moreover, where a diagnosis seems almost certain (for example, MRI highly suggestive of glioblastoma), this is explained to the patient upfront with clear indications of expected, realistic outcomes for that condition, including timelines (the honest conversation about reasonable competing options). This information is critical since they need to be able to make an informed decision about further treatment. Decision grids listing these options (see enclosed) are given to the patients after the consultation to facilitate the conveyance and understanding of complex and potentially overwhelming information.

After the discussion at the end of the consultation, patients make a decision along with their clinical team for one of the treatment options that works best for them based on what matters most to them. Patients are offered additional time to consider this at home with friends and family if they would prefer, but, in our experience, the vast majority make a decision in the first clinic visit. If they decide for a surgical option, a consent form is signed in clinic. An information leaflet with detailed information about the selected treatment option is given to the patients (e.g., “craniotomy,” “awake craniotomy,” “biopsy”) together with the decision grid and the contact number of the neuro-oncology CNS team.

In case the patients are not able to be supported to make a decision during the consultation, they are given the information leaflets, and they will communicate the decision as soon as it is reached. Depending on individual needs, a further consultation is scheduled that can be face-to-face, via video, or telephone.

## Discussion

Patient satisfaction with the SDM process was analyzed using the CollaboRATE score [2, 3, 12]. Patients reported high satisfaction already before the introduction of SDM with a mean score of 25.82 from a possible 27 points. This increased non-significantly to a mean score of 26.27 after team training and the introduction of SDM (*p*-value = 0.23). However, certain conversation techniques were introduced before the project started at baseline. A multivariate analysis including factors such as tumor type (HGG, LGG, metastasis) and gender did not show a statistically significant difference.

Interestingly, a significant improvement was seen among staff following the introduction of SDM despite some initial skepticism about the process and its overall benefits. Staff satisfaction was analyzed using the AquA questionnaire and values went from 61.68 to 90.95% (*p*-value < 0.001). The biggest absolute increase was seen among questions in Sect. 2, focusing on how much self-management of patients and SDM is supported by the clinical team. Members of staff found SDM to be a rewarding and beneficial process.

This study is one of the first studies in the literature showing the significant positive impact of SDM team training and its introduction on the attitude of healthcare staff. To our knowledge, this is the first study investigating the impact of SDM on healthcare staff in brain cancer care. Individualized decision grids for use in patients with HGG, LGG, and metastases, showing the respective three most appropriate treatment options were developed during this study (Supplementary Tables 1 to 3) and ready for use in daily clinical practice.

Patient satisfaction improved non-significantly after team training and the introduction of SDM with a high level of satisfaction already before. This positive impact of SDM in brain tumor patients is consistent with results from previous studies focusing on the same topic. Sorensen von Essen et al. published a systematic review about SDM in HGG patients in 2020. They included four studies in their review, and the conclusion was that most patients appreciate a SDM approach and that appropriate patient information and involvement can increase patients’ well-being. The use of a patient decision aid helped to increase knowledge, to decrease uncertainty, and affected the treatment decision-making of HGG patients [40]. Díaz et al. analyzed, in 26 patients diagnosed with HGG, the correlation between the quality of information the patients received about their condition, treatment options and prognosis, and the level of anxiety they showed immediately after they have received this information. They could show that patients who wanted to know everything about their illness, those who showed better understanding of the information, and those who were more satisfied with the information they received had statistically significant lower anxiety levels [10]. A randomized controlled study by El-Jawahri et al. published in 2010 used a video as a supplement to the verbal description for patients with malignant glioma that had to decide about their end-of-life treatment choices. The options were life-prolonging care such as cardiopulmonary resuscitation and ventilation, basic care allowing hospitalization but not reanimation, and comfort care focusing on symptom relief only. Patients who watched the video after having heard the verbal description were more likely to prefer comfort care and were more certain of their decision when compared to patients only hearing the verbal description [11].

Data about the impact of SDM on healthcare professionals in the literature is sparse, as most of the research about SDM focuses on the well-being of patients. There is a paucity of literature regarding the impact of SDM on healthcare staff in neuro-oncology. Elwyn et al. hypothesized that the experience of supporting patients in the achievement of an informed decision may be intrinsically rewarding, but that clinicians may also find the effort involved emotionally and cognitively taxing, adding to their workload burden. They felt that the consequences of SDM on clinicians should be studied and better understood, to improve the whole process [13]. Another study analyzing surgical decision-making did show that surgeons did favor SDM in cases of limited evidence for a given treatment plan, when multiple treatment options exist, and if treatment options impact on patients’ lifestyle [39]. A recently published pilot study on the use of “incorpoRATE” as a measure of the physicians’ willingness to incorporate SDM into practice did show that physicians felt less comfortable acting on informed patient choices when there was known incongruence with their own recommendations [4].

This study was conducted as a single-center prospective cohort study and is, therefore, subject to all the limitations of data collection inherent in such a study design. Studies comparing scores for the measurement of SDM quality in patients have shown that CollaboRATE has a lower decision quality compared to other scores (SURE and SDM Process_4). SURE and SDM Process_4 did also better discriminate the use of decision grids compared to CollaboRATE [6]. In the pediatric outpatient setting, CollaboRATE was preferred by parents over SDM Q-9 and did correlate better with satisfaction. However, the authors of the study found the psychometrics of the questionnaire only to be borderline acceptable [19]. One recently published study assessing SDM in the community mental health setting did show a high internal consistency of the CollaboRATE score [9]. The score did also show adequate reliability and validity when translated in other languages such as Spanish [35].

Patient-reported measures using the CollaboRATE score did not show significant improvements in scores after the introduction of SDM. However, this may have been different if another score was used as an outcome measure. In this study, it may be that there was not a true baseline, in that at the outset the team had started investigating and reading about SDM and beginning to practice these methods. Furthermore, the simple questionnaire is probably not very sensitive as there remains a bias in that patients, who develop a good rapport with their clinicians, have a tendency to reflect this in high scores. Perhaps a more granular method of assessment with focus groups or semi-structured interviews with qualitative thematic analysis would give more insight and may reveal significant differences. Another important consideration for the interpretation of the results is that the neuro-oncology team in our unit only consists of 5 persons at any given time point who work together very closely and efficiently. Most likely, team training and the implementation of SDM would have been much more difficult in a larger and less engaged team.

Decision grids seem to facilitate the SDM process [14, 15, 23, 31, 38, 42–44]. We have developed three different decision grids for use in brain tumor patients that are now used as an integral component of our daily clinical practice. They are given to the patient in written form but are also used to structure the conversation. The information contained in the grids is based on our own outcomes, and the level of risk can be adjusted on an individual case basis. If someone wants to use the grids for their clinical practice, our grids can be taken as a template. The treatment options and level of risks can be changed or modified as desired. As these grids are customized, they are usually not validated, which may be a possible limitation. A prospective study would be helpful to analyze the impact of these grids on SDM in our institution and to internally validate the grids.

Evaluating the patients` values, beliefs, and what matters most to them consumes additional time. As a consequence, our clinic times for the first appointments have increased from 40 min before the introduction of SDM to 50 min since. However, we believe this time is well spent (for all the reasons listed above) and may actually make subsequent conversations more straightforward. In our unit, since the SARS-CoV-2 pandemic started, we have switched the post-operative conversations to virtual using video. This is made possible by having invested time upfront to have open and honest conversations about the likely diagnosis and prognosis, rather than this being a challenge with histology results a week after surgery.

SDM is a helpful and rewarding part of the work of healthcare staff. Taking this into consideration, our staff now undergoes regular theoretical and practical training in SDM. The meaning of SDM is becoming more and more important in our work environment and is being built into tools such as online patient pathways. A specific neuro-oncological pathway was developed on a digital app “MyMedicalRecord” and is being implemented in our department currently. In the second part of this pathway, the patient’s feedback about the SDM process is obtained using the SDM-Q-9 questionnaire.

The effect of SDM on the theater caseload remains unknown. Studies have shown that the use of SDM leads to patients choosing more conservative options [11]. However, according to our own experience, SDM seems to lead to more conservative treatment decisions in some cases, particularly where, e.g., resection is not a given option and the impact of the remaining options on prognosis is limited (for example in glioblastoma where the outlook for treated biopsy only cases is very poor in our own audit data). The effect of SDM on the theater caseload is currently being investigated in a prospective study in our unit. Certainly, it has been our observation that slightly more patients are choosing less invasive interventional treatments now than they did prior to the introduction of SDM, albeit it was not our main driver for its introduction.

Studies have suggested that SDM has the potential to reduce complaints and litigation as it enhances communication and the relationship with the patients [13, 34]. Further research is needed to clarify these questions, but given the new GMC guidelines on informed consent in the UK and embracing SDM, it is clear that good conversations, adequately documented, will eliminate one medicolegal risk.

## Conclusions

Shared decision-making in a neuro-oncology setting is described in detail, along with 3 decision-support tools for the use in patients with HGG, LGG, and metastases. Team training in SDM and the introduction of techniques into daily practice can help to increase the understanding and satisfaction of clinicians, and both patients and carers report very high levels of satisfaction using the CollaboRATE questionnaires. Given the recent GMC guidelines and our experience to date, SDM should be routinely embedded as the standard of care in all neuro-oncology services.

## Supplementary information

Below is the link to the electronic supplementary material.

Supplementary file1 (DOCX 23 KB)

Supplementary file2 (DOCX 23 KB)

Supplementary file3 (DOCX 24 KB)

## Data Availability

The data that support the findings of this study are available from the corresponding author, SL, upon reasonable request.

## Abbreviations

ANOVA: Analysis of variance
AquA: Advancing Quality Alliance
BMC: Best Medical Care
CCT: Certificate of Completion Of Training
CNS: Clinical nurse specialist
CQUIN: Commissioning for Quality and Innovation
FAQs: Frequently asked questions
GMC: General Medical Council
HGG: High-grade glioma
KPS: Karnofsky performance score
LGG: Low-grade glioma
MAGIC: Making Good Decisions in Collaboration
MDT: Multidisciplinary Team
NHS: National Health System
NICE: National Institute for Health and Care Excellence
NMC: Nursing and Midwifery Council
PDSA: Plan Do Study Act
SDM: Shared decision making
UK: United Kingdom
US: United States
