# Supplementary material for: A prospective study of shared decision-making in brain tumor surgery
Source: Acta Neurochir (Wien). 2022 Dec 28;165(1):15–25. doi: 10.1007/s00701-022-05451-z (PMC9795149; doi:10.1007/s00701-022-05451-z)
Supplement: Supplementary file 1 — Supplementary file1 (DOCX 23 KB) [file 701_2022_5451_MOESM1_ESM.docx]

| **Treatment Options for Patients with a Suspected High Grade Glioma** | | | |
| --- | --- | --- | --- |
| **Frequently asked questions** | | | |
|  | **Best Medical Care** | **Biopsy**  **(+/- chemo/radiotherapy)** | **Craniotomy & Tumour Removal**  **(+/-chemo/radiotherapy)** |
| **For whom does this work best?** | Patients who do not wish to have invasive treatment with possible side effects & risks.  Patients with significant disability or who are not independent because of their tumour  Patients who have major risk factors for surgery.  Patients who’d rather focus on symptom control rather than surgery | Patients who wish to have active treatment or want to know the precise diagnosis.  Patients who don’t want the risks or side effects of major surgery.  Patients with a tumour that cannot be removed safely. | Patients with tumours that can be removed either mostly or completely.  Patients with headaches or symptoms due to the pressure of the tumour. |
| **What will this involve?** | Provide support and symptom control with medication to maintain quality of life. | A small day-case operation under local anaesthetic & sedation to obtain a sample of the tumour with a needle (+/- chemo or radiotherapy afterwards). | Admission to hospital for a more major operation to remove all/most of the tumour (+/- chemo or radiotherapy afterwards). |
| **What are the advantages?** | Avoids the risks and side effects of invasive treatment.  Focus is largely on controlling symptoms. | Achieves a diagnosis to plan further care and give information on prognosis.  Some patients find knowing a definitive diagnosis to be beneficial | Achieves a diagnosis.  Should improve symptoms due to pressure of the tumour.  Should improve overall survival by several months and may improve quality of life. |
| **What are the disadvantages?** | Life expectancy is more likely to be shorter than with treatment. | Life expectancy may be slightly shorter than with tumour resection.  Quality of life will be impaired while undergoing radiotherapy or chemotherapy.  Doesn’t reduce symptoms due to pressure of the tumour. | Quality of life will be impaired during the few weeks after surgery and during radiotherapy or chemotherapy. |
| **What are the risks?** | Little immediate risk. | 1 in 100 risk of major complication such as stroke/bleeding/death | 10 in 100 overall complication risk:  1-2 in 100 risk of stroke  1-2 in 100 risk of infection  1-2 in 100 risk of bleeding  <1 in 100 risk of death |

**Supplementary Table 1** Detailed information about treatment options craniotomy and tumour removal (+/- chemo/radiotherapy), biopsy (+/-chemo/radiotherapy) and BMC is listed in parallel.

From: A prospective study of shared decision making in brain tumor surgery, *Acta Neurochirurgica,* Leu S, Cahill J, Grundy PL, Department of Neurosurgery, Wessex Neurological Centre, University Hospital Southampton, Southampton, Hampshire, United Kingdom, severina.leu@unibas.ch
