# Supplementary material for: A prospective study of shared decision-making in brain tumor surgery
Source: Acta Neurochir (Wien). 2022 Dec 28;165(1):15–25. doi: 10.1007/s00701-022-05451-z (PMC9795149; doi:10.1007/s00701-022-05451-z)
Supplement: Supplementary file 2 — Supplementary file2 (DOCX 23 KB) [file 701_2022_5451_MOESM2_ESM.docx]

| **Treatment Options for Patients with a Suspected Low Grade Glioma** | | | |
| --- | --- | --- | --- |
| **Frequently asked questions** | | | |
|  | **Active Surveillance** | **Biopsy** | **Craniotomy & Tumour Removal** |
| **For whom does this treatment work best?** | Patients who have no symptoms.  Patients with small lesions.  Younger patients.  Patients who do not wish to have invasive treatments with possible side effects & risks. | Patients who want a diagnosis and/or wish to have active treatment.  Patients who don’t want the risks or side effects of major neurosurgery.  Patients with a tumour that cannot be safely removed. | Patients with tumours that can be removed either mostly or completely.  Patients with headaches or symptoms due to the pressure of the tumour. |
| **What will this**  **involve?** | Regular MRI scans to check for growth or change of the lesion. | A small day-case operation under local anaesthetic and sedation to obtain a sample of the tumour with a needle. | Admission to hospital for a more major operation to remove as much tumour as safely possible. |
| **What are the**  **advantages?** | Avoids the risks and side effects of invasive treatment.  Focus is largely on symptom control. | Achieves a diagnosis to plan further care and give information on prognosis.  Avoids an overnight stay in hospital. | Achieves a diagnosis.  Should improve any symptoms due to pressure of the tumour.  If you have seizures these may be better controlled after surgery.  May improve overall prognosis. |
| **What are the**  **disadvantages?** | If the lesion is a low grade glioma it is likely to grow/change with time. | Doesn’t reduce symptoms due to pressure of the tumour.  Will not help to control seizures.  Life expectancy may be shorter than if the tumour were completely/mostly removed. | Quality of life may be impaired for up to 6 weeks after surgery whilst recovering.  Requires on overnight stay in hospital |
| **What are the risks?** | Little immediate risk.  It might grow and cause symptoms over time. | 1 in 100 risk of major complications such as stroke/bleeding/death. | 10 in 100 overall complication risk:  1-2 in 100 risk of stroke  1-2 in 100 risk of infection  1-2 in 100 risk of bleeding  < 1 in 100 risk of death |

**Supplementary Table 2** Detailed information about treatment options craniotomy and tumour removal, biopsy and active surveillance is listed in parallel.

From: A prospective study of shared decision making in brain tumor surgery, *Acta Neurochirurgica,* Leu S, Cahill J, Grundy PL, Department of Neurosurgery, Wessex Neurological Centre, University Hospital Southampton, Southampton, Hampshire, United Kingdom, severina.leu@unibas.ch
