# Supplementary material for: A prospective study of shared decision-making in brain tumor surgery
Source: Acta Neurochir (Wien). 2022 Dec 28;165(1):15–25. doi: 10.1007/s00701-022-05451-z (PMC9795149; doi:10.1007/s00701-022-05451-z)
Supplement: Supplementary file 3 — Supplementary file3 (DOCX 24 KB) [file 701_2022_5451_MOESM3_ESM.docx]

| **Treatment Options for Patients with Suspected Brain Metastases** | | | |
| --- | --- | --- | --- |
| **Frequently asked questions** | | | |
|  | **Best Medical Care** | **Stereotactic Radiosurgery/Radiotherapy** | **Craniotomy & Tumour Removal** |
| **For whom does this work best?** | Patients who do not wish to have invasive treatment with possible side effects & risks.  Patients with significant disability or who are not independent because of their disease.  Patients who have major risk factors for surgery. | Patients with a previously diagnosed cancer.  Patients who don’t want the risks or side effects of neurosurgery.  Patients with smaller tumours under 3cm.  Patients with multiple tumours (total volume below 20ml)  Patients with deep or difficult to remove tumours | Patients in whom the diagnosis is uncertain or have not had a biopsy before.  Patients with headaches or symptoms due to the pressure of the tumour or swelling.  Patients with larger tumours greater than 3cm  Patients with tumours that can be safely removed. |
| **What will this**  **involve?** | Provide support and symptom control with medication to maintain quality of life. | These are day-case treatments involving the precise delivery of a high dose of radiotherapy to the tumour:  Stereotactic Radiosurgery – a single dose.  Stereotactic Radiotherapy – 2-5 doses. | Admission to hospital for an operation to remove the tumour. |
| **What are the**  **advantages?** | Avoids the risks and side effects of invasive treatment.  Focus is largely on symptom control and quality of life. | In most patients overall prognosis is improved by several months (the same as with surgery).  Avoids the need & risks of neurosurgery.  Avoids overnight stay in hospital. | In most patients will improve overall prognosis by several months (the same as with SRS).  Will provide a precise diagnosis.  Will usually improve pressure symptoms if present and allow steroids to be stopped over a few days, improving quality of life. |
| **What are the**  **disadvantages?** | Life expectancy is more likely to be shorter. | Does not remove the tumour for diagnosis.  Swelling may not resolve as quickly as with surgery so may require a longer course of steroids.  Doesn’t reduce pressure effects immediately if present.  Late side effects can occur. | Quality of life may be impaired for a few weeks whilst recovering from surgery.  Requires an overnight stay in hospital |
| **What are the risks?** | Little immediate risk | 10 in 100 risk of swelling that may require further courses of steroids or rarely surgery to remove the tumour  1 in 3 risk of tumour continuing to grow or growing back | 10 in 100 overall complication risk:  1-2 in 100 risk of stroke  1-2 in 100 risk of infection  1-2 in 100 risk of bleeding  Less than 1 in 100 risk of death  1 in 2 risk of tumour growing back |

**Supplementary Table 3** Detailed information about treatment options craniotomy and tumour removal, stereotactic radiosurgery/-therapy and BMC is listed in parallel.

From: A prospective study of shared decision making in brain tumor surgery, *Acta Neurochirurgica,* Leu S, Cahill J, Grundy PL, Department of Neurosurgery, Wessex Neurological Centre, University Hospital Southampton, Southampton, Hampshire, United Kingdom, severina.leu@unibas.ch
